# Genome Sequences of Novel Members of Previously Described DNA and RNA Virus Families, Isolated from Feces of a Drill Monkey in Nigeria

**DOI:** 10.1128/MRA.00092-20

**Published:** 2020-04-23

**Authors:** U. George, C. Simsek, T. O. C. Faleye, O. Arowolo, A. Oragwa, O. M. Adewumi, J. Matthijnssens, J. A. Adeniji

**Affiliations:** a Department of Biological Sciences, Redeemer’s University, Ede, Nigeria; b Department of Microbiology, Immunology, and Transplantation, KU Leuven, Leuven, Belgium; c Laboratory of Viral Metagenomics, Rega Institute, KU Leuven, Leuven, Belgium; d Department of Virology, Faculty of Basic Medical Sciences, College of Medicine, University of Ibadan, Ibadan, Nigeria; e Center for Human Virology and Genomics, Microbiology Department, Nigerian Institute of Medical Research, Lagos, Nigeria; f Viral Vaccines Unit, National Veterinary Research Institute, Vom, Nigeria; g Department of Veterinary Microbiology and Pathology, University of Jos, Jos, Nigeria; h Infectious Disease Institute, College of Medicine, University of Ibadan, Ibadan, Nigeria; i WHO National Polio Laboratory, University of Ibadan, Ibadan, Nigeria; Queens College

## Abstract

The genomes of seven novel members of previously described DNA and RNA virus families are described here. These viruses were recovered using a viral metagenomic approach from the stool of a drill monkey (Mandrillus leucophaeus) housed in a sanctuary in Cross River State, Nigeria.

## ANNOUNCEMENT

Zoonotic transmission of viruses between nonhuman primates (NHPs) and humans and its impact on the emergence and reemergence of viral pathogens have been well documented (1–3). Despite such studies, we are not aware of any metagenomic exploration of virus diversity among NHPs in Nigeria. Hence, in an effort to explore virus diversity among NHPs in Nigeria, we performed a metagenomic survey of virus diversity in a stool sample from a drill monkey (Mandrillus leucophaeus) in a sanctuary in the South South region of the country. Drills are among Africa’s endangered mammals (because their current population in the wild may be <10,000) and are listed on the high conservation priority list by the International Union for Conservation of Nature (4). Here, we describe the genomes of novel members of previously described DNA and RNA virus families in a stool sample from a drill monkey in Nigeria.

The fresh fecal sample analyzed in this study was collected from the floor of an encampment housing drill monkeys in the Afi Mountain Wildlife Sanctuary in Cross River State, Nigeria. The sample was resuspended (1:9) in phosphate-buffered saline and subsequently subjected to the NetoVIR protocol (5). Briefly, after homogenization and centrifugation at 17,000 × *g* for 3 min, the stool suspension was filtered (0.8-μm filter), and the filtrate was treated with nucleases. The treated stool suspension was then subjected to nucleic acid extraction using a Qiagen total nucleic acid extraction kit, and both RNA and DNA were randomly amplified using the whole-transcriptome amplification kit 2 (Sigma-Aldrich). Library preparation was subsequently done using the Nextera XT DNA kit. The library was then subjected to paired-end sequencing (2 × 150 bp) using the NextSeq platform (Illumina). Trimming was done using Trimmomatic (6), while assembly was done using SPAdes (7) and MEGAHIT (8). DIAMOND (9) was used to annotate the contigs obtained using the NCBI nonredundant protein database as the reference. The output was displayed using KronaTools to facilitate viral contig identification. All software was used with default settings.

Seven near-complete genomes of novel members of previously described DNA and RNA virus families were recovered (Table 1), representing 150,956 (1.79%) of the 8,416,538 reads generated. The three DNA viruses are members of the families *Parvoviridae* (*Protoparvovirus* and *Ambidensovirus*) and *Circoviridae* (*Cyclovirus*). Three of the four RNA viruses are members of the *Picornaviridae* (*Cosavirus*, *Enterovirus*, and *Sapelovirus*), while the fourth is a member of the *Dicistroviridae* (*Aparavirus*). Unlike the other five genomes, the *Aparavirus* (*Dicistroviridae*) and *Ambidensovirus* (*Parvoviridae*) representatives might be insect viruses and are most likely relics from the diet of the drills (Table 1).

**TABLE 1:** Assembly and characterization details of the novel genomes described in this study

Family	Genus (genome type)	Species^*a*^	GenBank accession no.	Genome size (nucleotides)	Coverage (fold)	No. (%) of mapped reads^*b*^	GC content (%)	Novelty type	Criterion for novelty^*c*^	GenBank accession no. for most similar sequence(s) (% similarity)^*d*^	Likely host
*Picornaviridae*	*Enterovirus* (RNA)	*Enterovirus J*	MN265403	7,282	39.48	2,294 (0.03)	44	Serotype	<88% aa sequence identity in VP1	AHY21607.1 (64.04)	Mammal
*Picornaviridae*	*Sapelovirus* (RNA)	*Sapelovirus C*^*e*^	MN784122	8,207	182.64	12,036 (0.14)	39	Species	<70% aa sequence identity in complete polyprotein	ACG63545 (59.15)	Mammal
*Picornaviridae*	*Cosavirus* (RNA)	*Cosavirus G*^*e*^	MN784123	7,714	798.03	49,316 (0.59)	47	Species	<65% aa sequence identity in P1	FJ438907 (43.59)	Mammal
*Dicistroviridae*	*Aparavirus* (RNA)	ABPV complex	MN296283	9,533	187.16	14,189 (0.17)	39	Strain	Not yet established	NP_851403.1 (78.79 [NS]); NP_851404.2 (83.27 [SP])	Insect
*Parvoviridae*	*Protoparvovirus* (DNA)	BokiPPvirus^*e*^	MN265404	4,310	94.59	3,285 (0.04)	42	Species	<85% aa sequence identity in NS1	QDI06029.1 (71.50)	Mammal
*Parvoviridae*	*Ambidensovirus* (DNA)	BokiADvirus^*e*^	MN784124	5,260	58.99	2,529 (0.03)	37	Species	<85% aa sequence identity in NS1	NP_046813.1 (71.93)	Insect
*Circoviridae*	*Cyclovirus* (DNA)	BokiCvirus^*e*^	MN784125	1,800	4,604.92	67,307 (0.8)	49	Species	<80% genome-wide nucleotide sequence identity	GQ404854 (67.82)	Mammal

a ABPV, acute bee paralysis virus; BokiPPvirus, Boki Afi Mountain protoparvovirus; BokiADvirus, Boki Afi Mountain ambidensovirus; BokiCvirus, Boki Afi Mountain cyclovirus.

b The total number of reads generated was 8,416,538.

c aa, amino acid; NS, nonstructural protein; SP, structural protein. Assemblies were primarily done using SPAdes. MEGAHIT assembly was done to resolve unclear assemblies (genome organizations) recovered from SPAdes (e.g., SPAdes assembled *Sapelovirus C* with two open reading frames, while MEGAHIT assembled it with one; the two were of similar lengths, however).

d All similarity values are amino acid similarities (determined using BLASTp) except for the value for the *Cyclovirus* entry, which is nucleotide similarity (determined using BLASTn).

e Likely new species.

The genomes of seven novel members of previously described DNA and RNA virus families found in the stool of a drill monkey in Cross River State, Nigeria, are described here. A number of novel partial genomes were also found (data not shown). Here, we show that, by conserving drills, we might unknowingly have also conserved a previously undescribed virome. Whether these viruses are pathogenic in drills or might have zoonotic potential remains to be investigated. Current efforts are directed at better exploring virus diversity in this conserved ecosystem using a noninvasive approach, and our preliminary data suggest that these viruses are circulating.

### Data availability.

The genomes described here have been deposited under SRA number PRJNA599206 and GenBank accession numbers MN265403, MN265404, MN296283, and MN784122 to MN784125.
